# Supplementary material for: MANF regulates hypothalamic control of food intake and body weight
Source: Nat Commun. 2017 Sep 18;8:579. doi: 10.1038/s41467-017-00750-x (PMC5603516; doi:10.1038/s41467-017-00750-x)
Supplement: Supplementary file 1 — Supplementary Information [file 41467_2017_750_MOESM1_ESM.pdf]

Description of Supplementary Files

File Name: Supplementary Information
Description: Supplementary Figures

File Name: Peer Review File

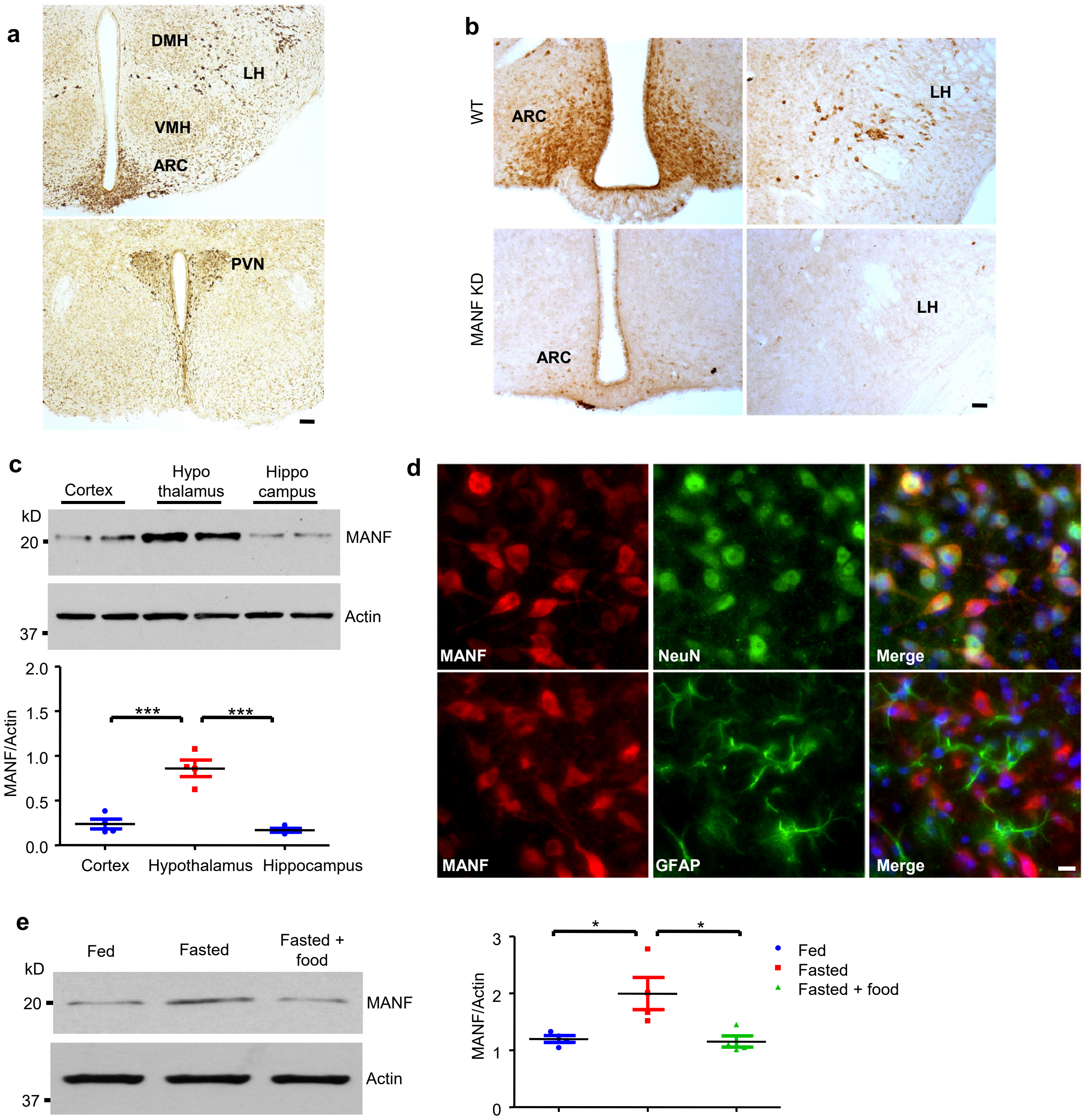

Supplementary Figure 1 Characterization of MANF in the hypothalamus (related to Figure 1). (a) Low magnification (5 X) images showing the expression of MANF in the hypothalamus of wild type (WT) mice (ARC: arcuate nucleus; LH: lateral hypothalamus; VMH: ventromedial hypothalamus; DMH: dorsomedial hypothalamus; PVN: paraventricular nucleus; Scale bar: 100 μm). **(b)** Immunohistochemistry staining of MANF expression in the hypothalamus of WT and MANF knockdown (MANF KD) mice using another MANF antibody (Scale bar: 50 μm). **(c)** Western blotting and quantitative analysis of MANF expression in different brain regions of WT mice (*** $P < 0.001$, n = 4, one-way ANOVA with Tukey post-tests, F = 36.81, $P < 0.0001$). **(d)** Double immunostaining of the hypothalamus of wild type mice using MANF antibody together with NeuN or GFAP antibody (Scale bar: 20 μm). **(e)** Wild type mice were fasted for 48 hours, or fasted for 48 hours and then fed with regular diet for 4 hours. Western blotting and quantitative analysis was performed to check MANF expression in the hypothalamus (* $P < 0.05$, n = 4, one-way ANOVA with Tukey post-tests, F = 7.198, $P = 0.0136$).

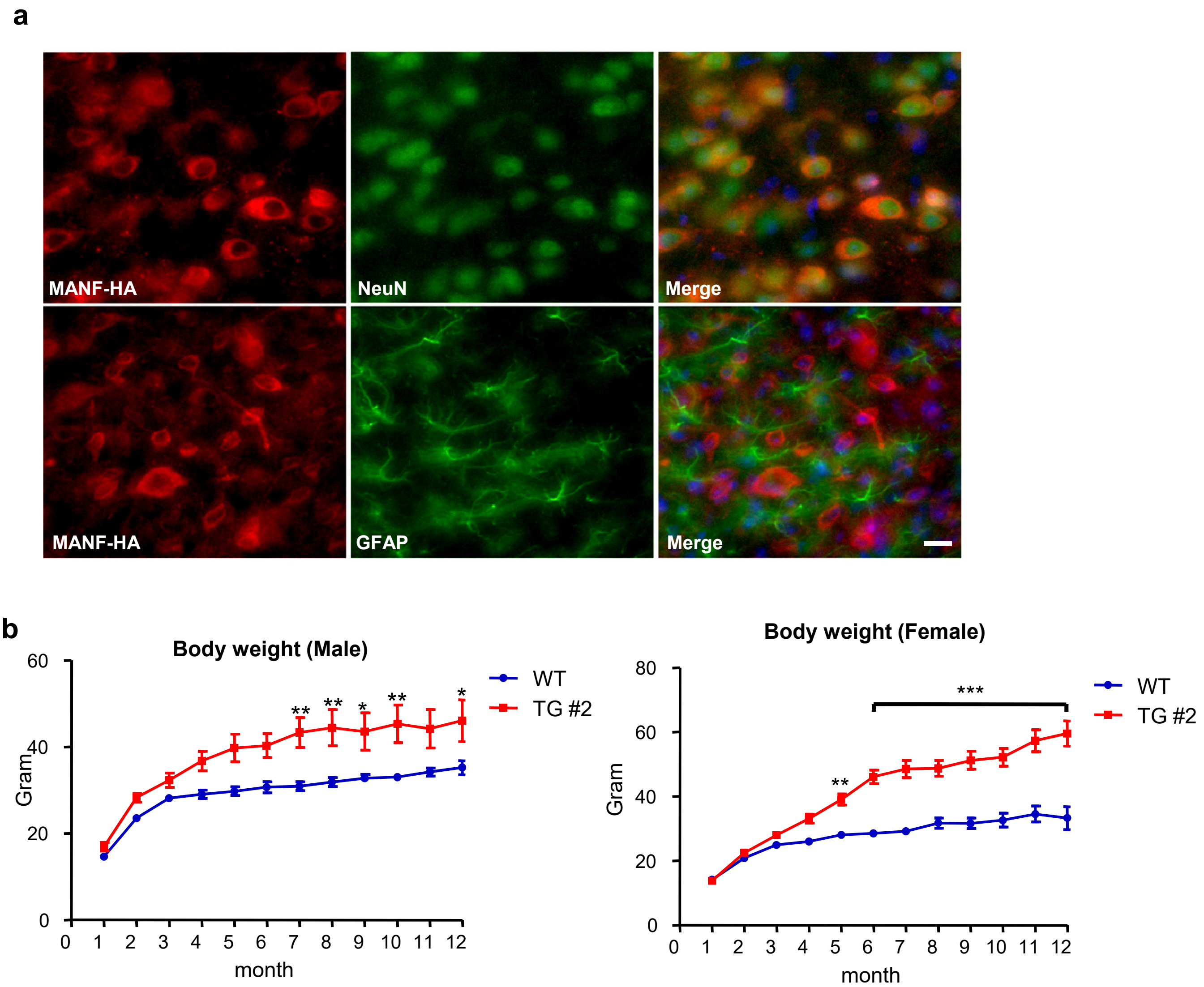

Supplementary Figure 2 Characterization of MANF transgenic mouse model (related to Figure 2). (a) Double immunostaining of the hypothalamus of MANF transgenic mice using HA antibody together with NeuN or GFAP antibody (Scale bar: 20 μm). **(b)** Body weights of another MANF transgenic mouse line (TG #2) were measured monthly from 1-month to 12-month old. Starting from 5-month old, both male and female TG mice showed significantly increased body weight compared with wild type (WT) littermates (* $P < 0.01$, ** $P < 0.01$, *** $P < 0.001$, n = 5, two-way ANOVA with Bonferroni post-tests, Male, F = 77.25, $P < 0.0001$; Female, F = 274.8, $P < 0.0001$). Data are represented as mean ± SEM.

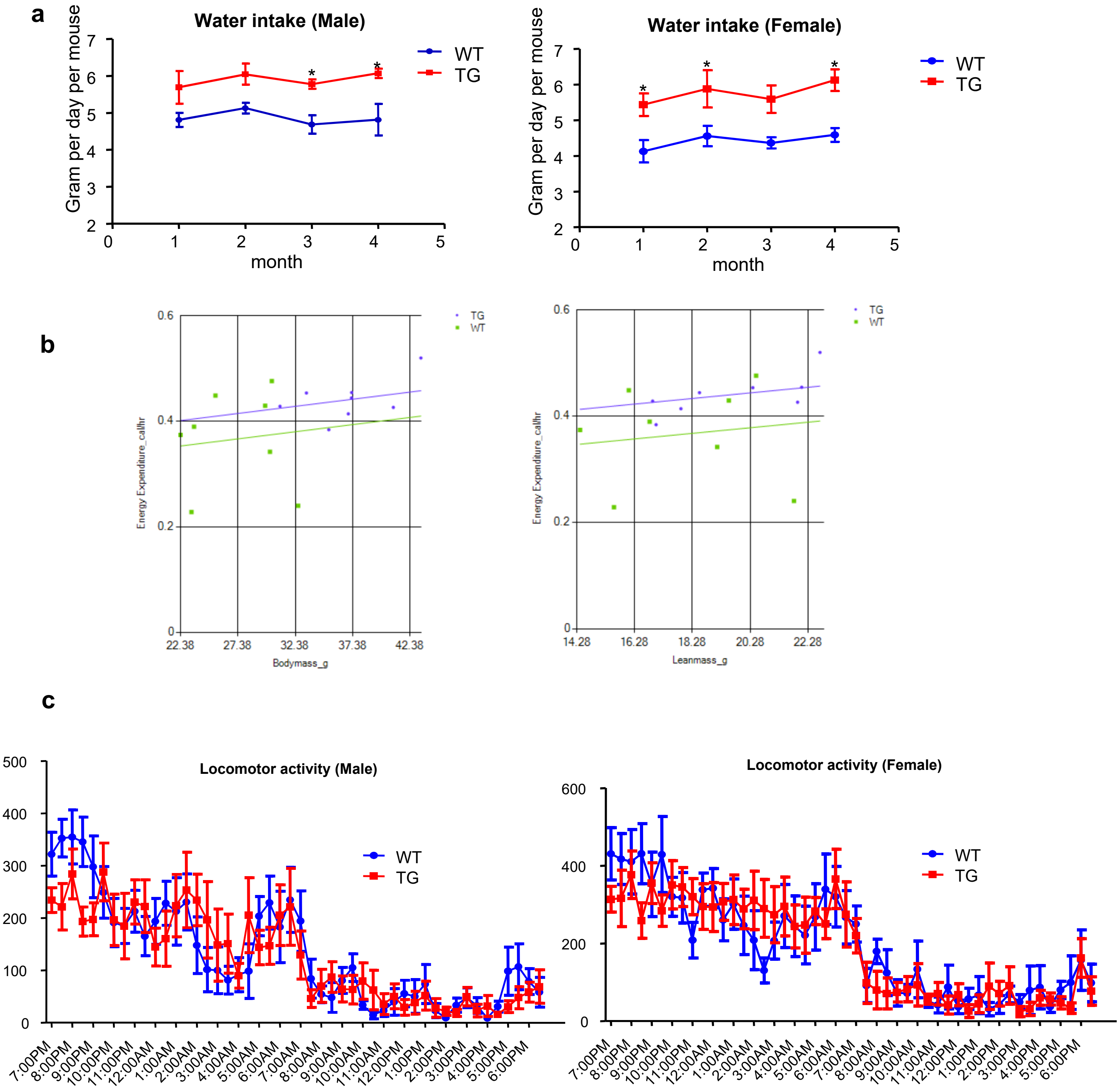

Supplementary Figure 3 Further characterization of MANF transgenic mouse model (related to Figure 3). (a) Daily water intake of wild type (WT) and MANF transgenic (TG) mice were measured monthly from 1-month to 4-months of age (* $P < 0.05$, n = 6 - 7, two-way ANOVA with Bonferroni post-tests, Male, F = 26.64, $P < 0.0001$; Female, F = 31.86, $P < 0.0001$). **(b)** ANCOVA statistical analysis indicates the interaction between energy expenditure and body weight is not significant (n = 8, for total body mass, $P = 0.6171$; for lean body mass, $P = 0.446$), nor is the difference of energy expenditure between different genotypes (For total body mass, $P = 0.438$; for lean body mass, $P = 0.1108$). **(c)** Locomotor activity of 2-month old WT and TG mice in a 24-hour period (n = 9 - 10, two-way ANOVA with Bonferroni post-tests, Male, F = 1.157, $P = 0.2823$; Female, F = 0.4497, $P = 0.5027$; dark period is from 7:00 PM to 7:00 AM, light period is from 7:00 AM to 7:00 PM). Data are represented as mean ± SEM.

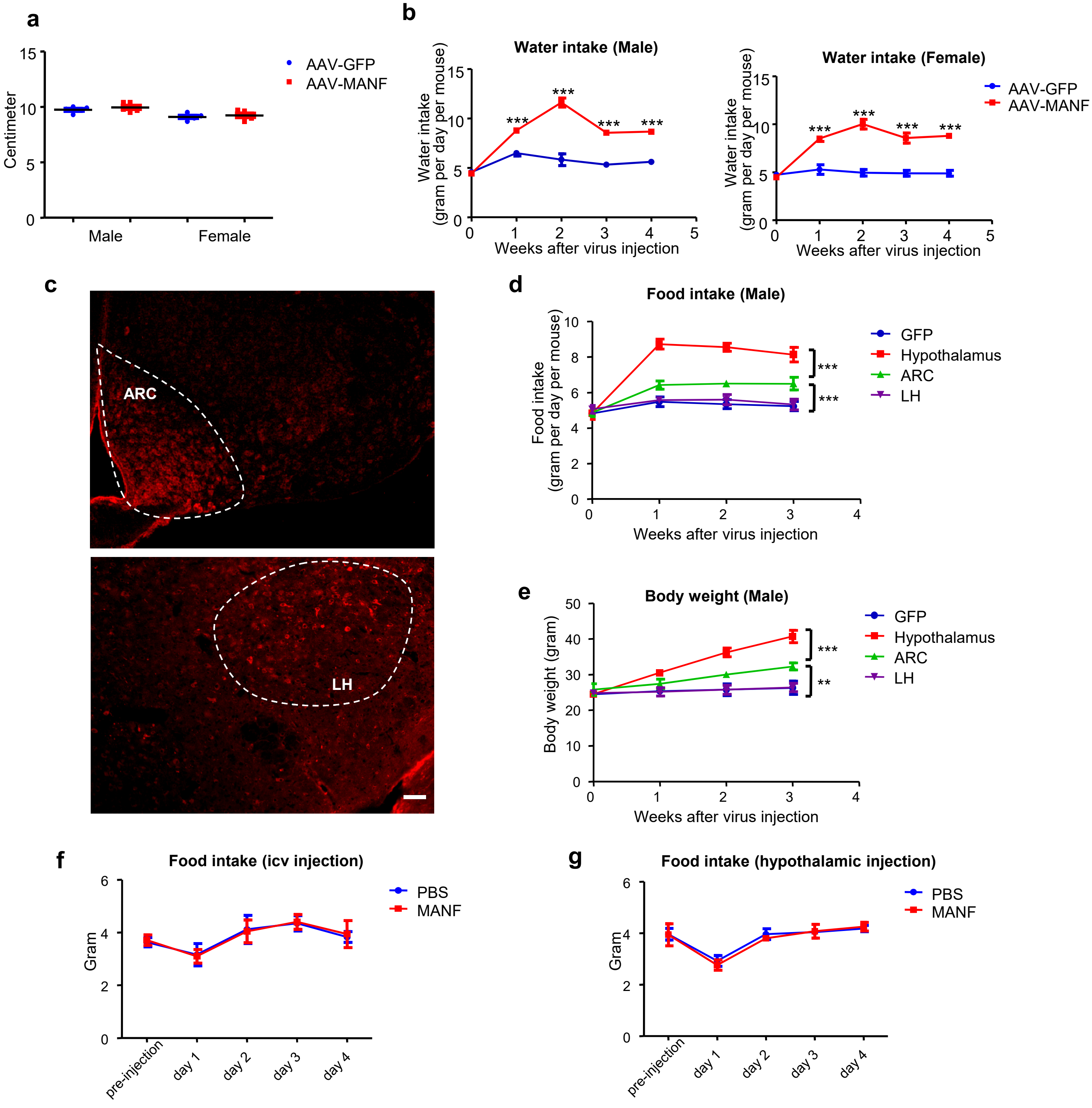

Supplementary Figure 4 Characterization of wild type mice injected with AAV-MANF (related to Figure 4). (a) Snout-anus lengths of male and female wild type (WT) mice 4 weeks after injected with AAV-GFP or AAV-MANF (n = 5, student t test, Male, t = 0.9918, *P* = 0.3503; Female, t = 0.6163, *P* = 0.5548). **(b)** Water intake of male and female mice were measured weekly from before AAV-GFP or AAV-MANF injection to 4 weeks after injection (*** *P* < 0.001, n = 5, two-way ANOVA with Bonferroni post-tests, Male, F = 250.7, *P* < 0.0001; Female, F = 191.4, *P* < 0.0001). **(c)** Immunofluorescent staining with HA antibody shows the preferential expression of AAV-MANF in the arcuate nucleus (ARC) or lateral hypothalamus (LH) of injected mice (Scale bar: 100 μm). **(d)** Food intake of 2-month old WT mice infected with AAV-MANF in the whole hypothalamus (Hypothalamus), specifically in the ARC (ARC) or specifically in the LH (LH) was measured weekly. Mice injected with AAV-GFP were controls (*** *P* < 0.001, n = 5, two-way ANOVA, ARC and GFP, F = 19.96, *P* < 0.0001; Hypothalamus and ARC, F = 52.55, *P* < 0.0001). **(e)** Body weight of 2-month old WT mice infected with AAV-MANF in the whole hypothalamus (Hypothalamus), specifically in the ARC (ARC) or specifically in the LH (LH) was measured weekly. Mice injected with AAV-GFP were controls (** *P* < 0.01 *** *P* < 0.001, n = 5, two-way ANOVA, ARC and GFP, F = 12.89, *P* = 0.0012; Hypothalamus and ARC, F = 23.54, *P* < 0.0001). **(f)** Daily food intake of WT mice, which were injected with PBS or recombinant mouse MANF protein into the third ventricle, was measured from pre-injection till 4 days post-injection (n = 7, two-way ANOVA with Bonferroni post-tests, F = 0.05727, *P* = 0.8117). **(g)** Daily food intake of WT mice, which were injected with PBS or recombinant mouse MANF protein directly into the hypothalamus, was measured from pre-injection till 4 days post-injection (n = 5, two-way ANOVA, F = 0.1057, *P* = 0.747).

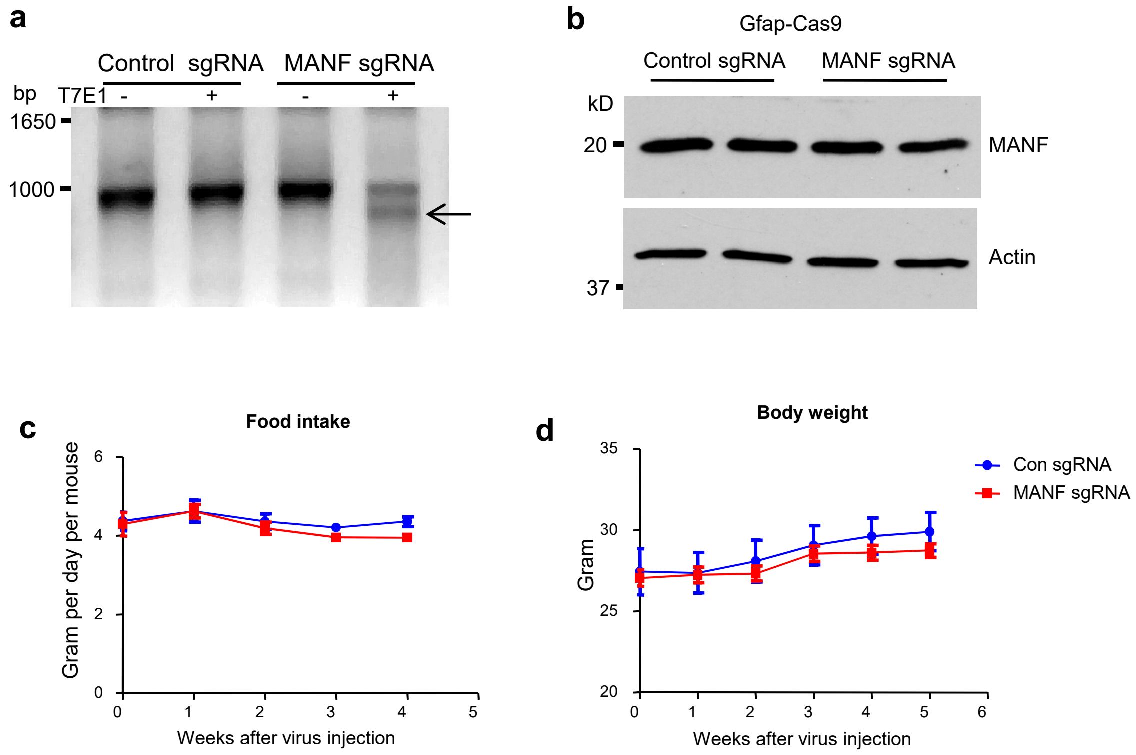

Supplementary Figure 5 Characterization of wild type mice with MANF knockdown (related to Figure 5). (a) T7 Endonuclease 1 (T7E1) digestion result of MANF genomic locus from N2A cells transfected with control sgRNA/CMV-Cas9 or MANF sgRNA/CMV-Cas9. Arrow indicates the cleaved products. **(b)** Western blotting result of MANF level in the hypothalamus of WT mice injected with AAV-control-sgRNA/AAV-Gfap-Cas9 or AAV-MANF-sgRNA/AAV-Gfap-Cas9. **(c)** Food intake of 3-month old male WT mice injected with AAV-control-sgRNA/AAV-Gfap-Cas9 or AAV-MANF-sgRNA/AAV-Gfap-Cas9 was measured weekly (n = 5, two-way ANOVA with Bonferroni post-tests, F = 2.257, P = 0.1486). **(d)** Body weight of 3-month old male WT mice injected with AAV-control-sgRNA/AAV-Gfap-Cas9 or AAV-MANF-sgRNA/AAV-Gfap-Cas9 was measured weekly (n = 5, two-way ANOVA with Bonferroni post-tests, F = 1.477, P = 0.2301).

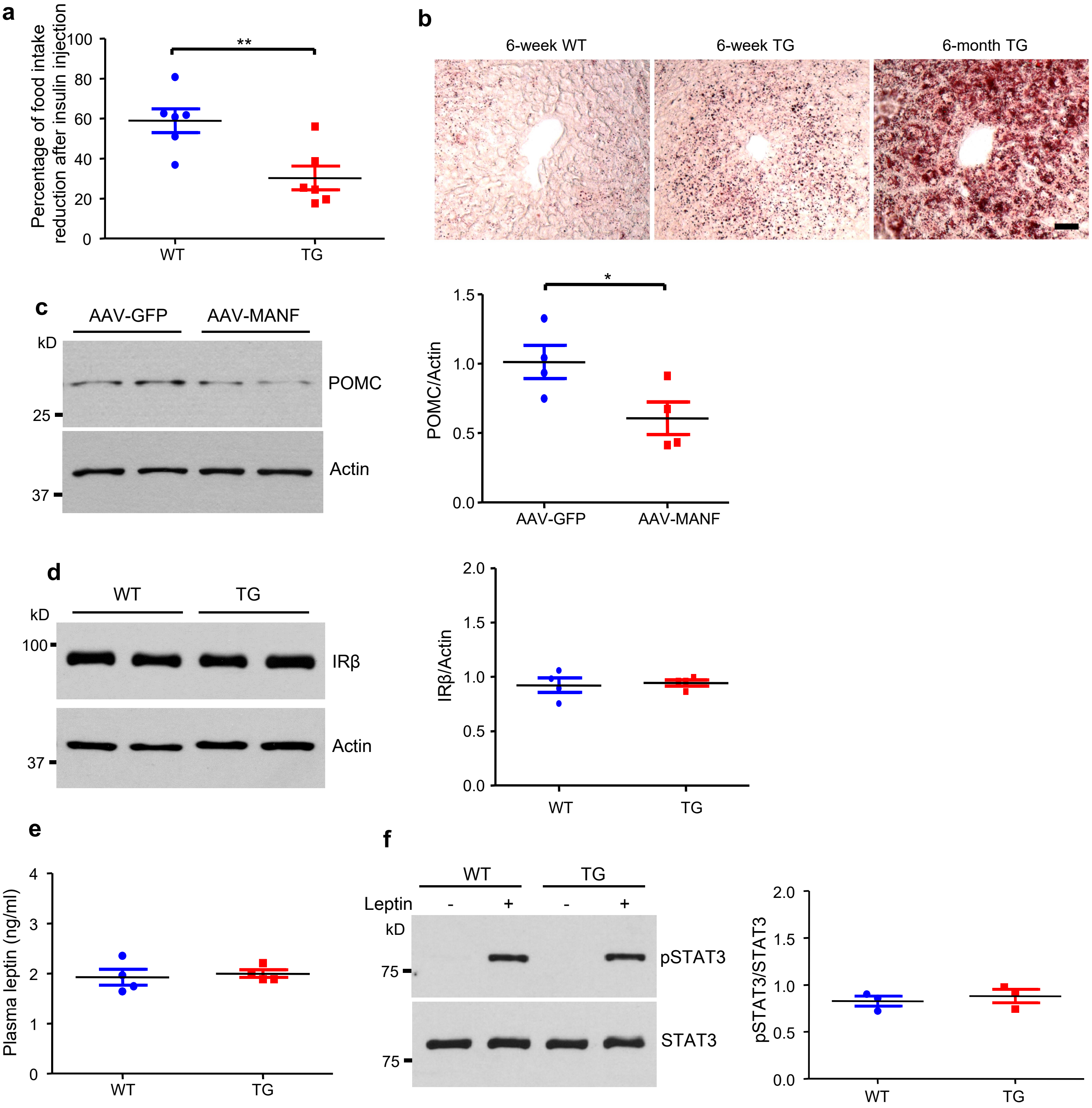

Supplementary Figure 6 Further characterization of insulin resistance in mice with MANF overexpression (related to Figure 6). (a) The percentage of food intake reduction 1 day after wild type (WT) and MANF transgenic (TG) mice were injected with insulin into the third ventricle (** $P < 0.01$, n = 6, student t test, t = 3.42, $P = 0.0066$). **(b)** Liver steatosis of differently aged WT and MANF TG mice was examined by Oil Red O staining. MANF TG mice had age-related accumulation of fat in the liver (Scale bar: 100 µm). **(c)** Western blotting and quantitative analysis of POMC levels in the hypothalamus of wild type mice injected with AAV-GFP or AAV-MANF (n = 4, student t test, t = 2.403, $P = 0.0265$). **(d)** Western blotting and quantitative analysis of IRβ expression in the hypothalamus of 2-month old WT and MANF TG mice (n = 4, student t test, t = 0.2527, $P = 0.4084$). **(e)** Plasma leptin levels of 6-week old WT and MANF TG mice were determined by ELISA (n = 4, student t test, t = 0.4057, $P = 0.699$). **(f)** Western blotting and quantitative analysis of pSTAT3 expression in the hypothalamus 6-week old WT and MANF TG mice with intraperitoneal leptin injection (n = 3, student t test, t = 0.6055, $P = 0.5775$).

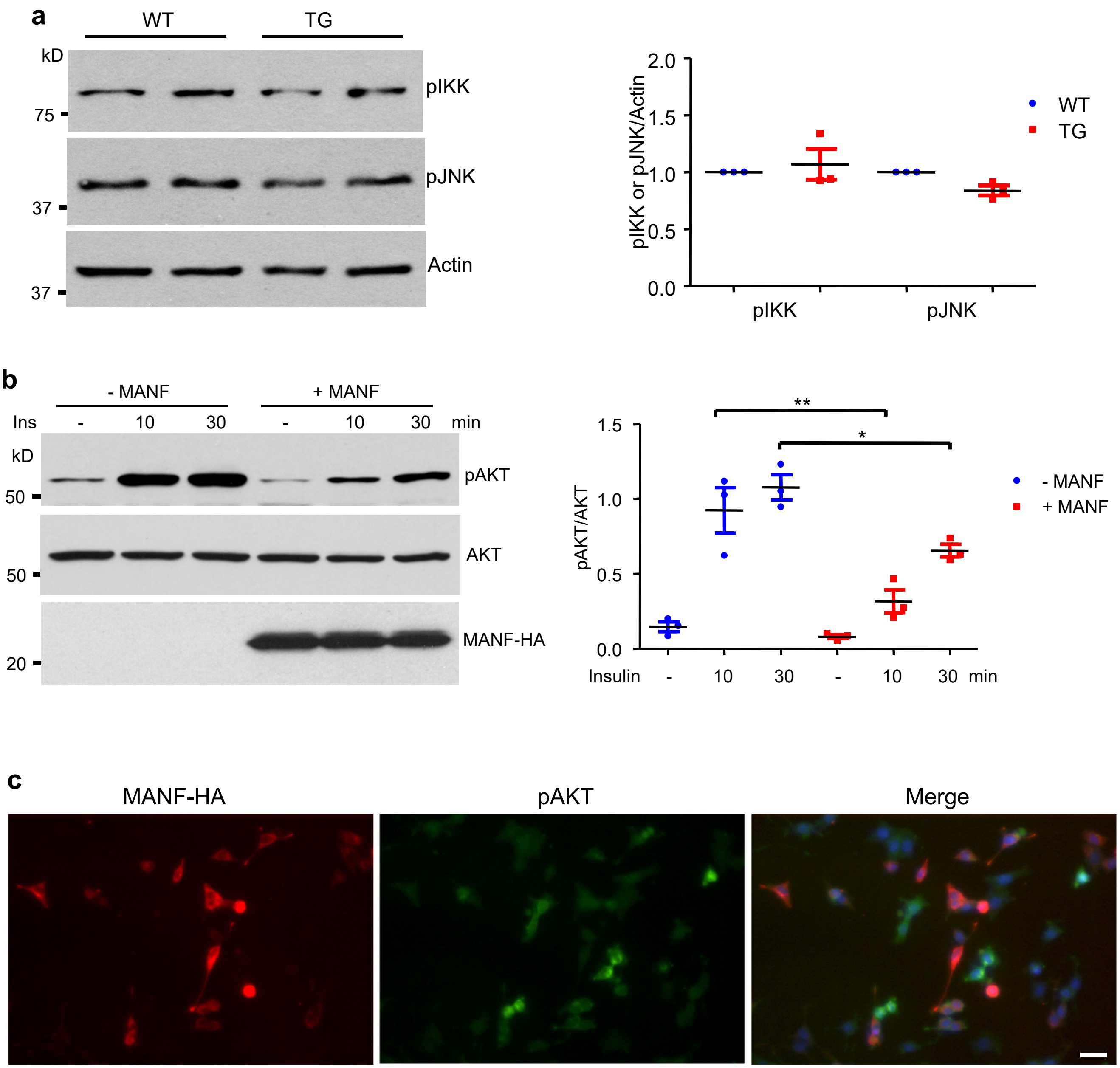

Supplementary Figure 7 MANF overexpression does not trigger inflammation (related to Figure 6). (a) Western blotting and quantitative analysis of pIKK and pJNK levels in the hypothalamus of 2-month old wild type and MANF TG mice (n = 3, student t test, pIKK, t = 1.352, P = 0.309; pJNK, t = 3.699, P = 0.066). **(b)** PC12 cells transfected with MANF-HA (+ MANF) or untransfected (- MANF) were treated with insulin (Ins) for different lengths of time: untreated (-), 10 minutes or 30 minutes. Western blotting and quantitative analysis was performed to reveal pAKT levels in PC12 cell lysates (* $P < 0.05$, ** $P < 0.01$, n = 3, one-way ANOVA with Tukey post-tests, F = 26.4, $P < 0.0001$). AKT served as loading controls. Data are represented as mean ± SEM. **(c)** PC12 cells were transfected with HA-MANF for 2 days, serum-starved for 4 hours, and treated with insulin for 10 minutes. Double immunostaining was performed to show HA-MANF expression and pAKT levels in each individual cell (Scale bar: 20 μm).

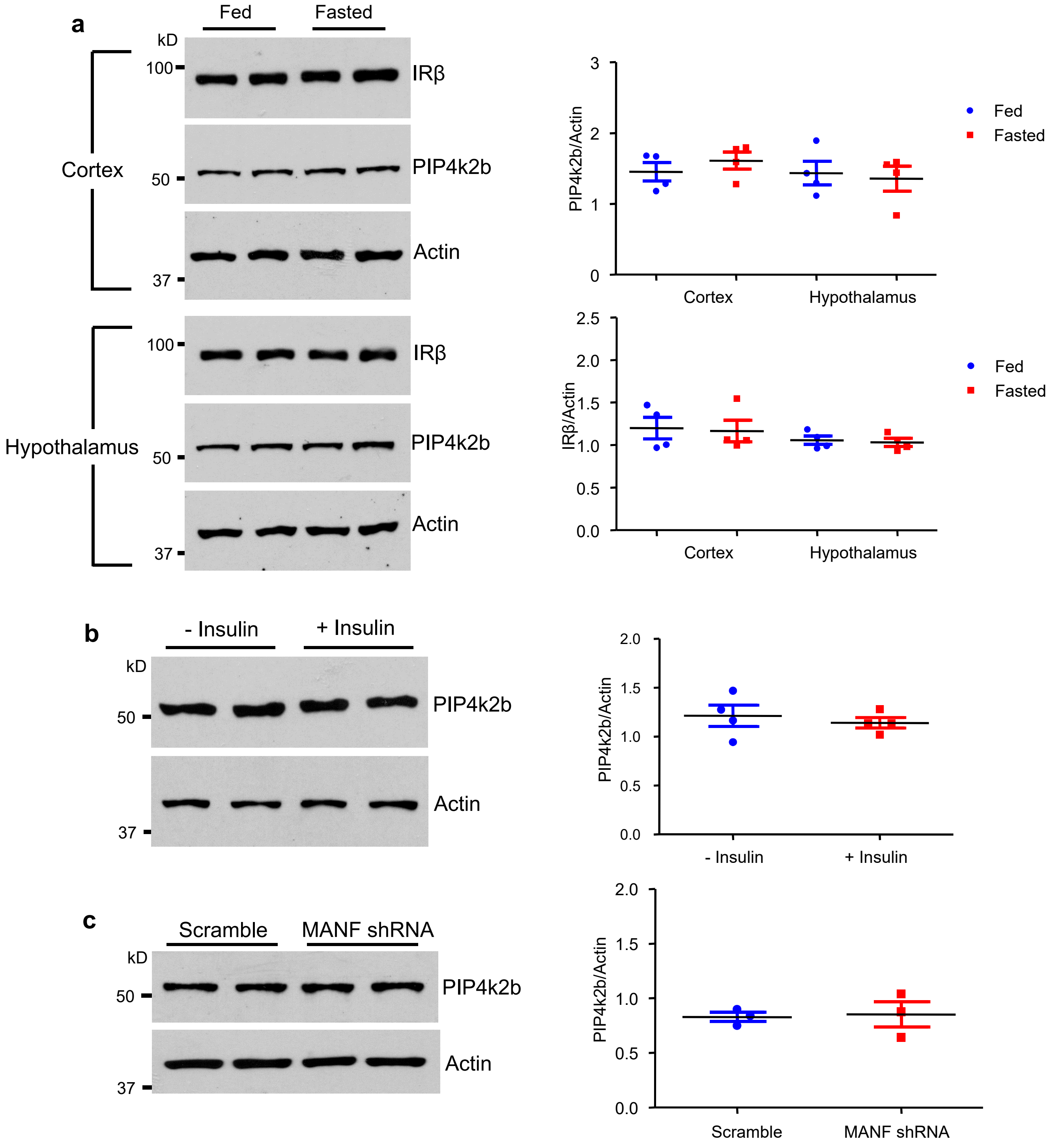

Supplementary Figure 8 Examination of PIP4k2b expression (related to Figure 7). (a) Western blotting and quantitative analysis of IRβ and PIP4k2b levels in the cortex and hypothalamus of WT mice fasted for 48 hours (n = 4, student t test, IRβ, t = 1.598, *P* = 0.1042; PIP4k2b, t = 0.8917, *P* = 0.4069). **(b)** Western blotting and quantitative analysis of PIP4k2b expression in the hypothalamus of MANF transgenic mice after intraperitoneal insulin injection (n = 4, student t test, t = 0.5816, *P* = 0.582). **(c)** Western blotting and quantitative analysis of PIP4k2b expression in the hypothalamus of WT mice injected with AAV-scramble shRNA or AAV-MANF-shRNA (n = 3, student t test, t = 0.1844, *P* = 0.8626).. Data are represented as mean ± SEM.

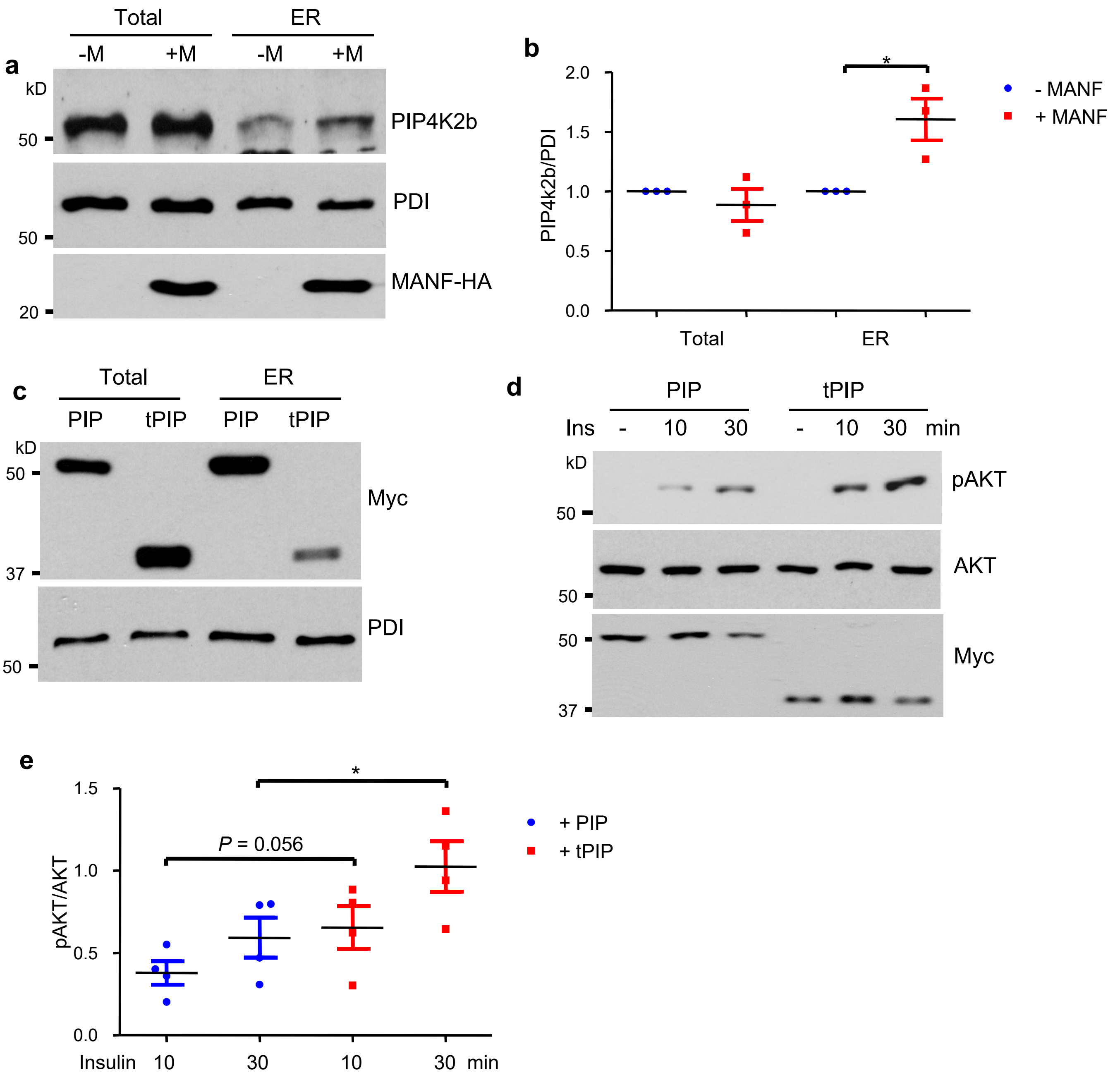

Supplementary Figure 9 PIP4k2b is localized in the ER and modulates insulin signaling (related to Figure 7). (a) Subcelluar fractionation was performed using PC12 cells transfected with HA-MANF (+ M) or untransfected (- M). Western blotting was used to show PIP4k2b levels in both total and ER fraction. **(b)** Quantification of western blotting results in Supplementary Figure 9a. PIP4k2b level was significantly increased in the ER fraction of PC12 cells transfected with HA-MANF (+ MANF), compared with untransfected cells (- MANF) (* $P < 0.05$, n = 3; student t test, $P = 0.0374$). **(c)** ER localization of full length PIP4k2b (PIP) and PIP4k2b with N-terminal truncation (tPIP) was compared. **(d)** PC12 cells were transfected with either full length PIP4k2b (PIP) or PIP4k2b with N-terminal truncation (tPIP) for 2 days, serum-starved for 4 hours, and treated with insulin for different periods of time. PIP4k2b with N-terminal truncation was not as effective as full length PIP4k2b in inhibiting insulin mediated AKT phosphorylation. Myc antibody was used to show PIP and tPIP expression. **(e)** Quantification of western blotting results in Supplementary Figure 9d. AKT phosphorylation level was significantly increased in cells transfected with tPIP, compared with those transfected PIP (* $P < 0.05$, n = 4; student t test, 10 minutes, t = 1.86, $P = 0.0561$; 30 minutes, t = 2.215, $P = 0.0343$). Data are represented as mean ± SEM.

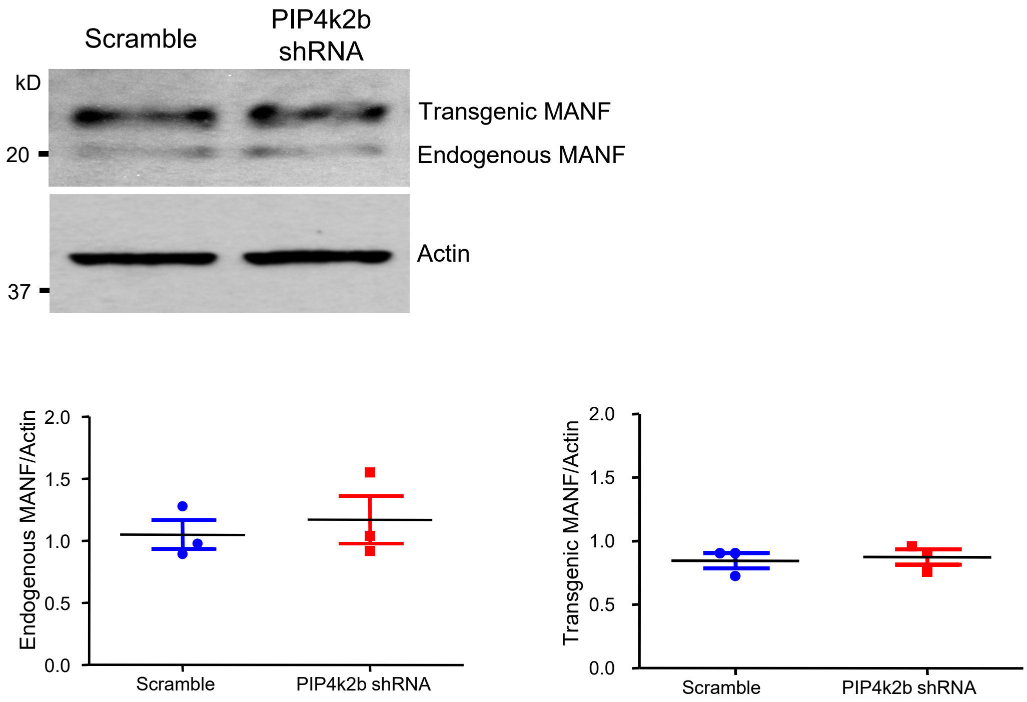

Supplementary Figure 10 MANF expression in PIP4k2b knockdown mice (related to Figure 8). Western blotting and quantitative analysis of endogenous and transgenic MANF expression in the hypothalamus of MANF transgenic mice injected with AAV-scramble-shRNA or AAV-PIP4k2b-shRNA (n = 3, student t test, endogenous, t = 0.528, *P* = 0.6254; transgenic, t = 0.3557, *P* = 0.7401). Data are represented as mean ± SEM.

Figure 1

b

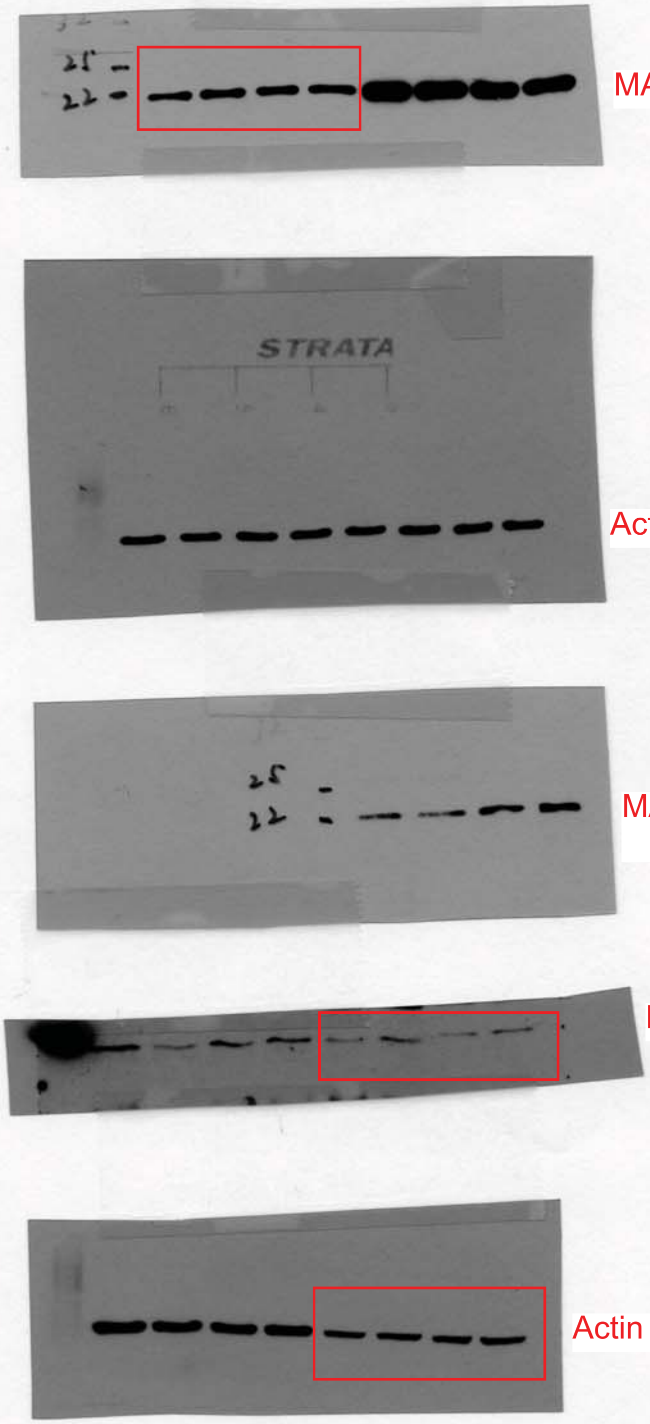

MANF cortex

Actin cortex and hypothalamus

MANF hypothalamus

MANF liver

Actin liver

Supplementary Figure 11 (continued)

Figure 2

b

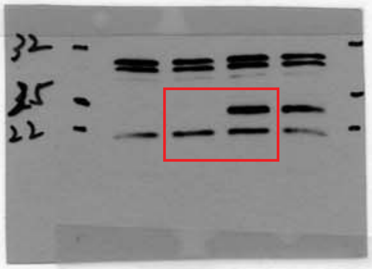

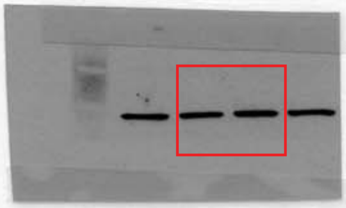

c

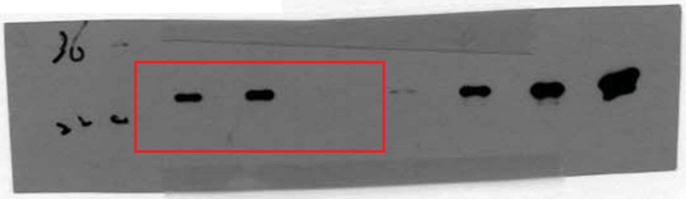

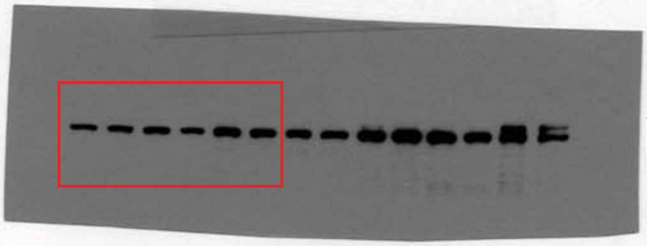

Supplementary Figure 11 (continued)

Figure 4

b

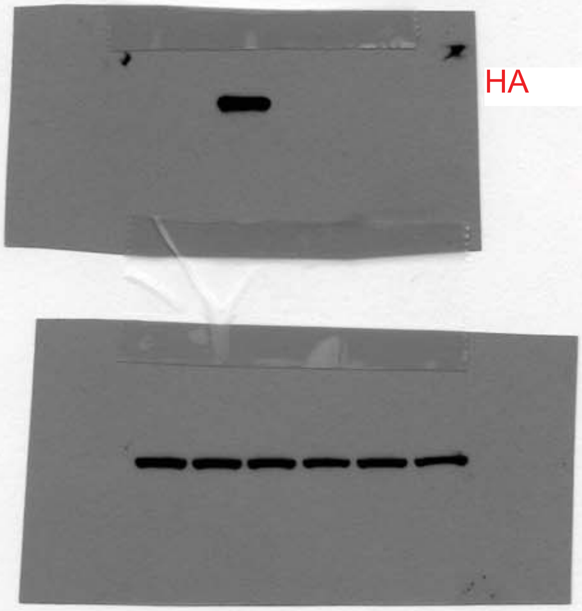

Supplementary Figure 11 (continued)

Figure 5

b

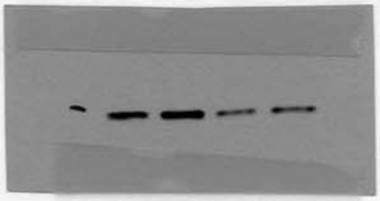

MANF

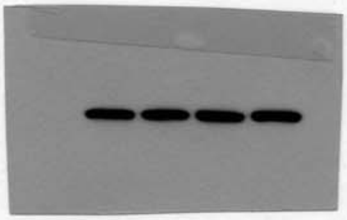

Actin

h

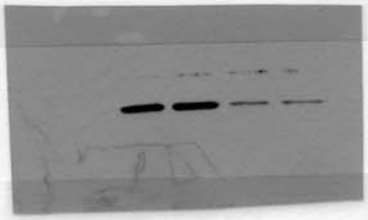

MANF

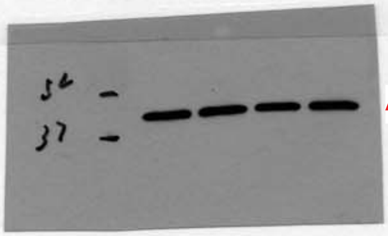

Actin

Supplementary Figure 11 (continued)

Figure 6

a

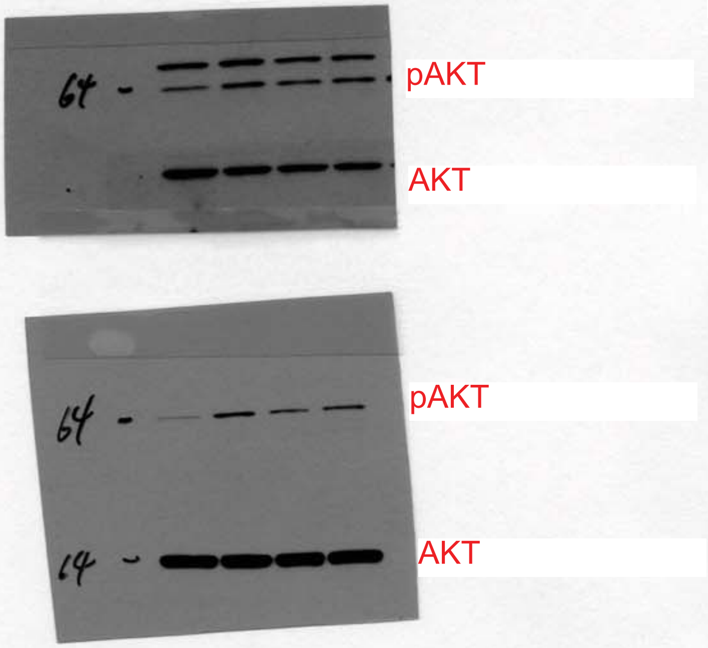

b

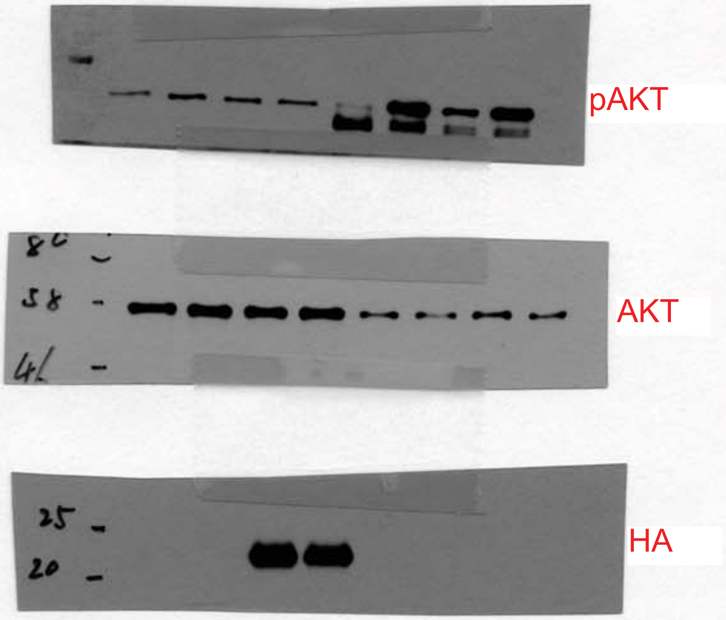

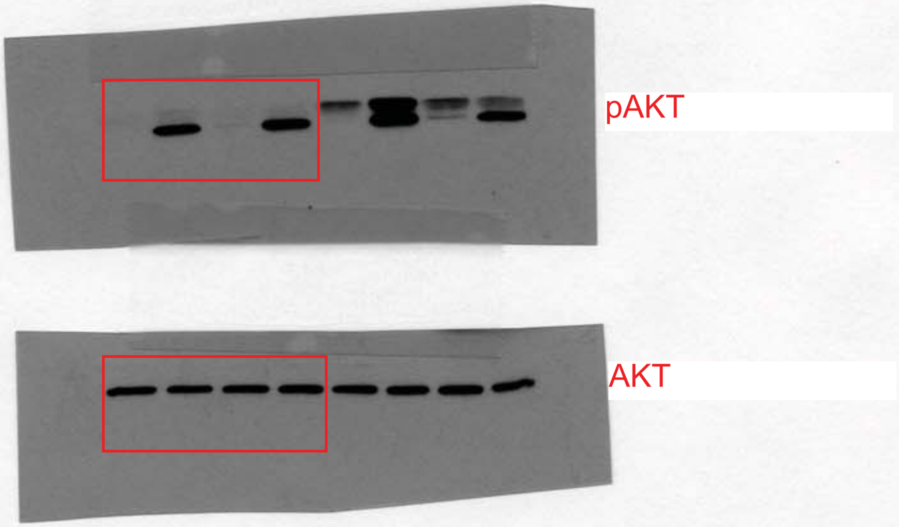

Supplementary Figure 11 (continued)

c

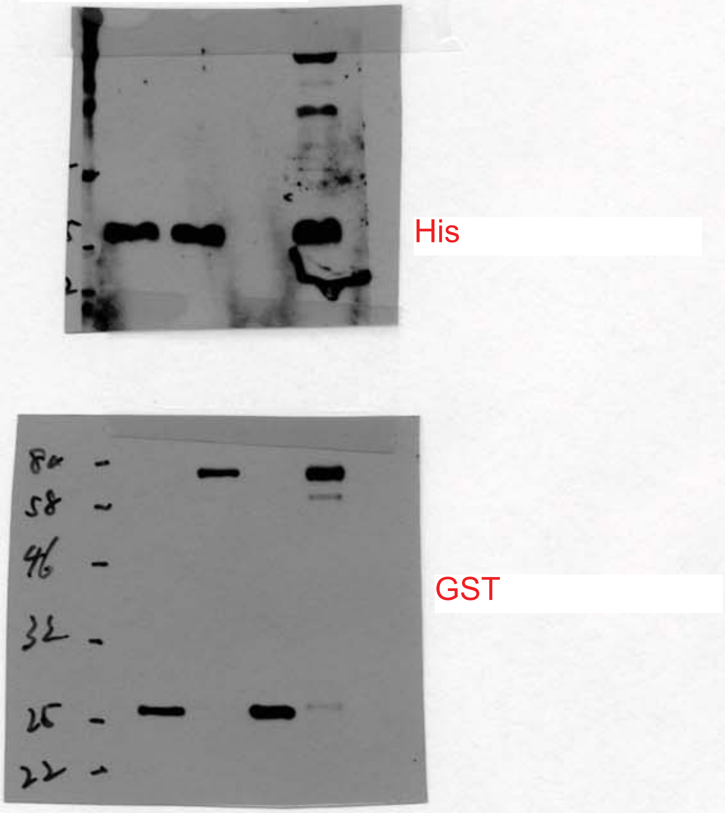

d

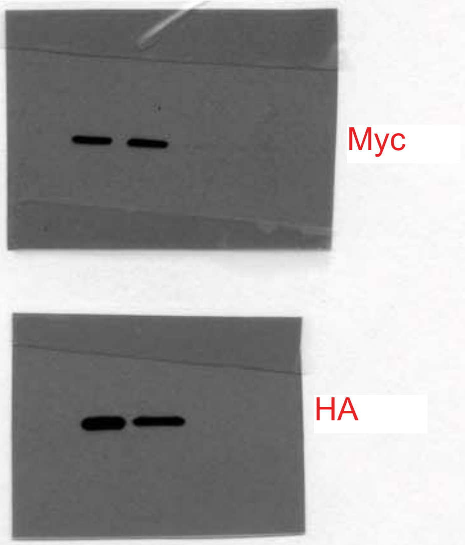

e

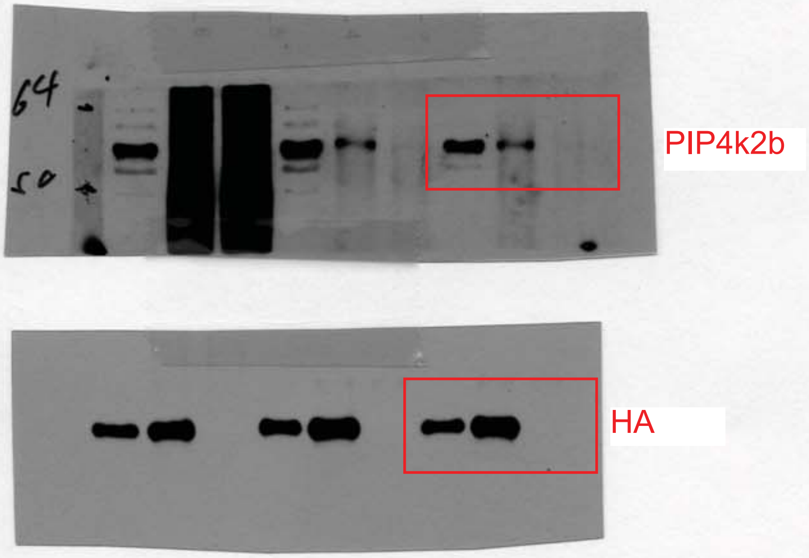

f

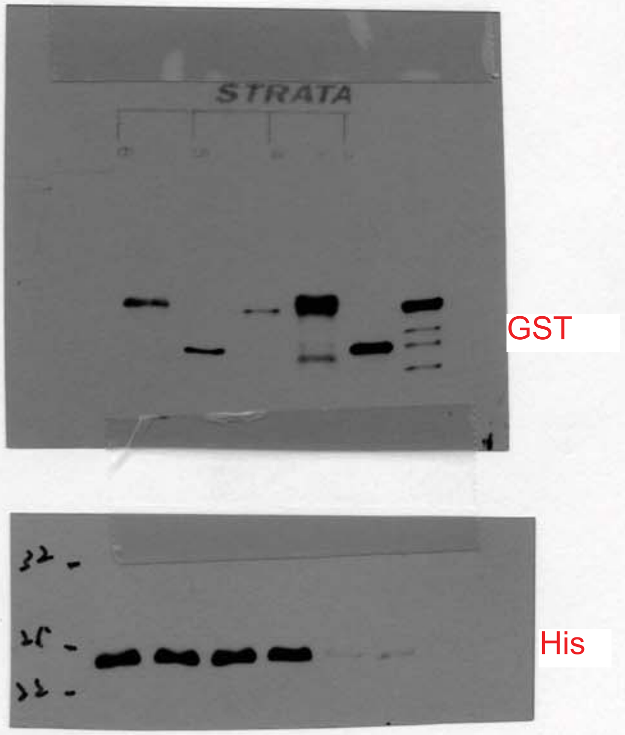

Supplementary Figure 11 (continued)

Figure 7

g

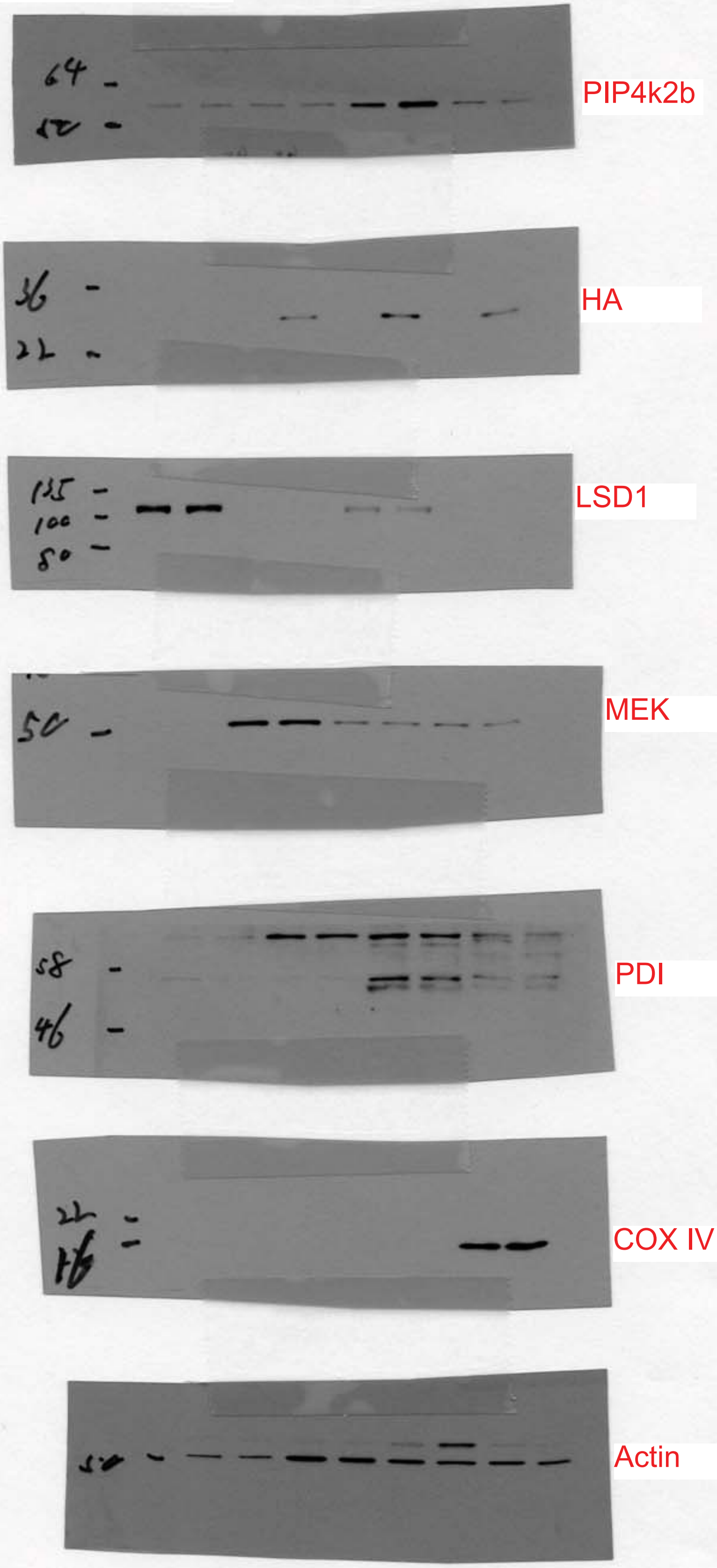

h

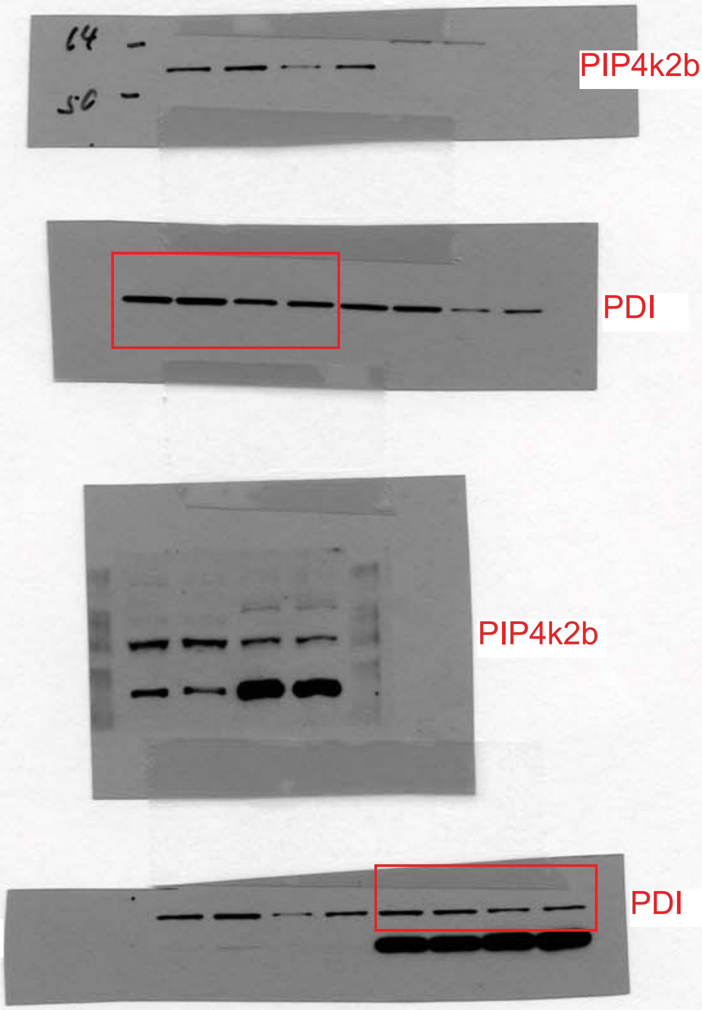

Supplementary Figure 11 (continued)

Figure 8

a

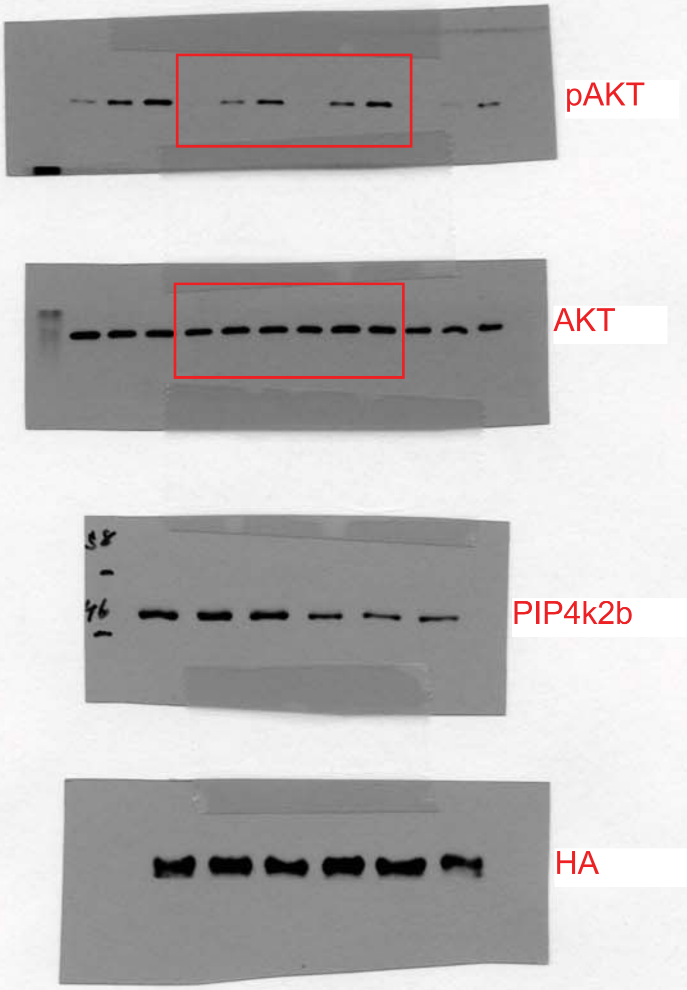

f

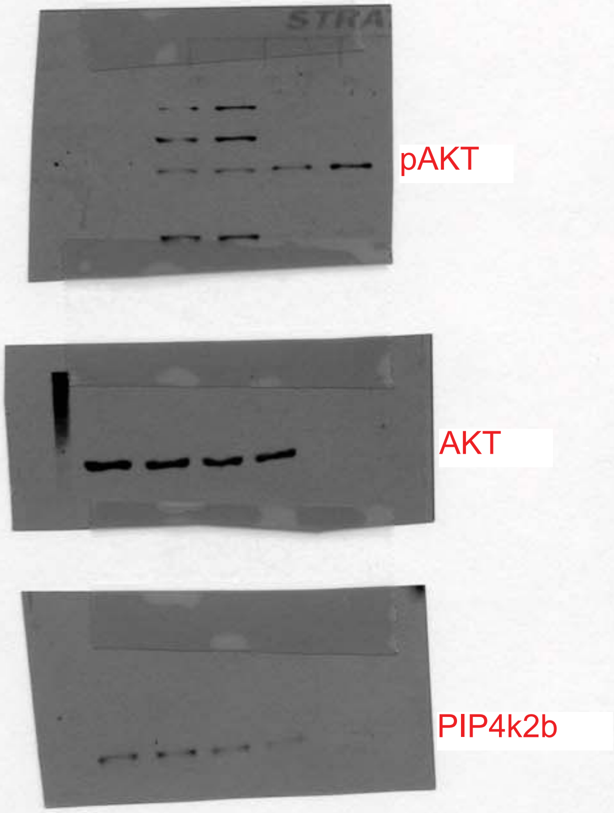

Supplementary Figure 11 (continued)

Supplementary Figure 1

c

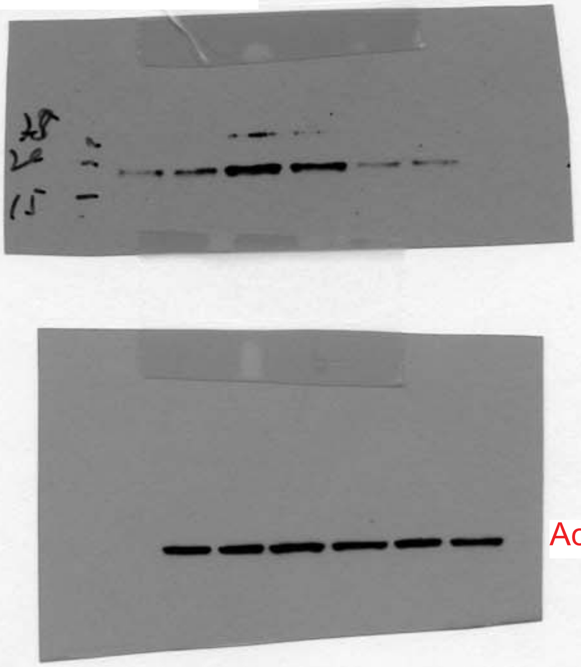

MANF

Actin

e

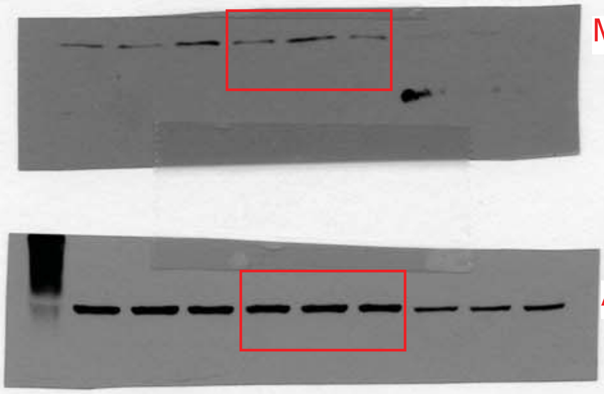

MANF

Actin

Supplementary Figure 11 (continued)

Supplementary Figure 5

b

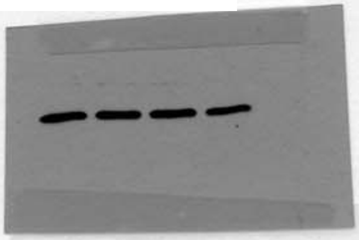

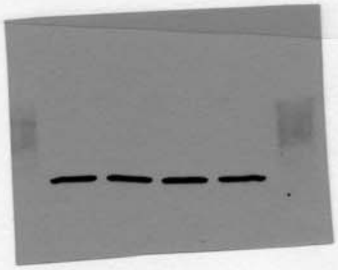

Supplementary Figure 11 (continued)

Supplementary Figure 6

c

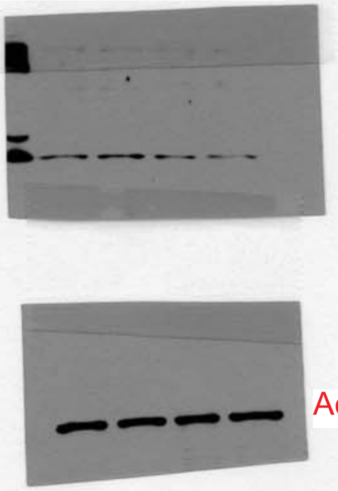

POMC

Actin

f

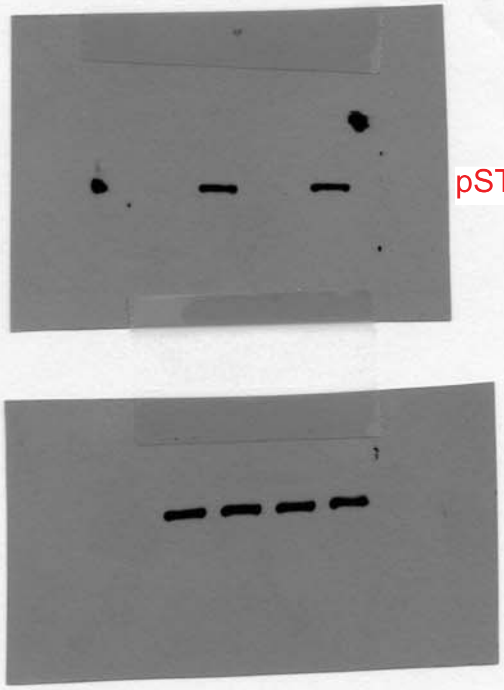

pSTAT3

STAT3

d

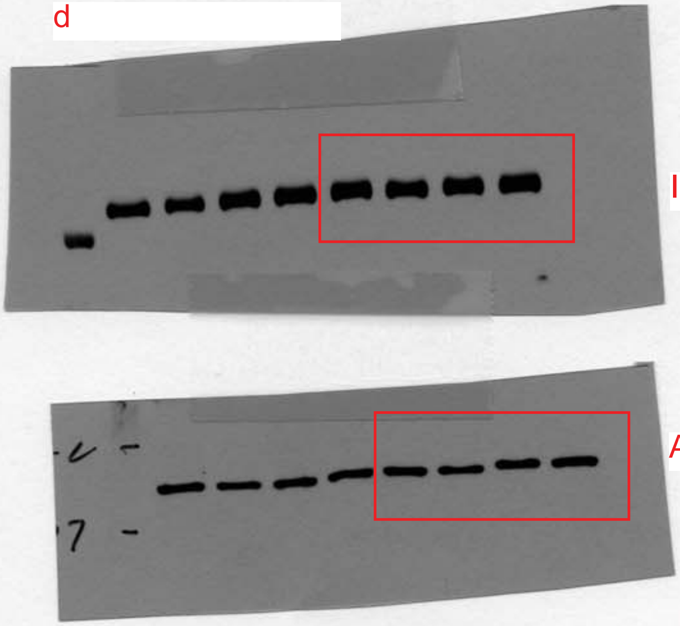

IRβ

Actin

Supplementary Figure 11 (continued)

Supplementary Figure 7

a

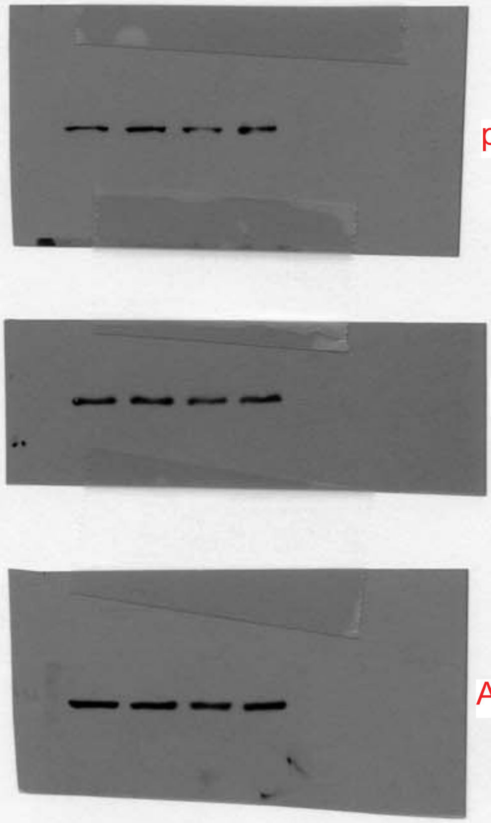

b

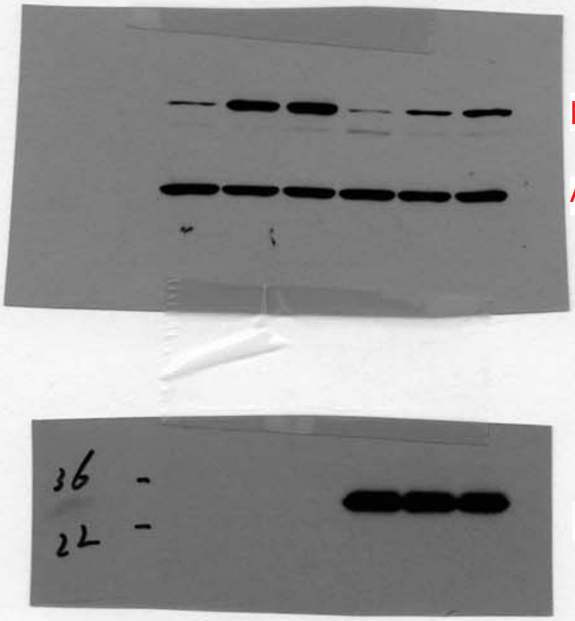

Supplementary Figure 11 (continued)

a

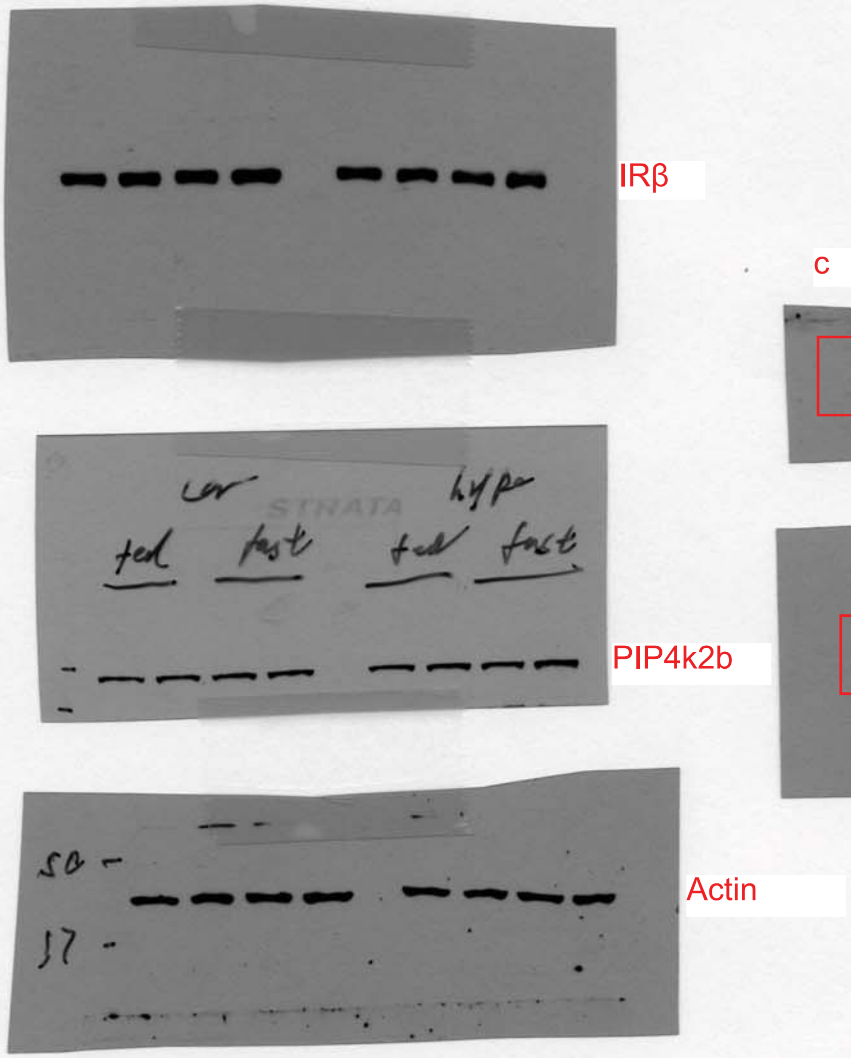

c

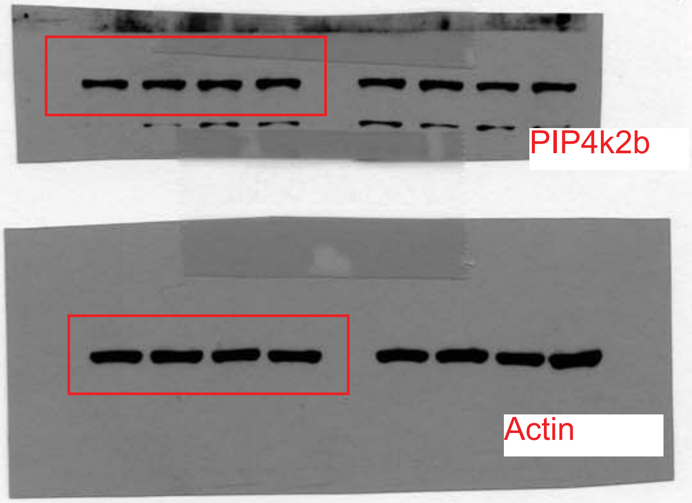

b

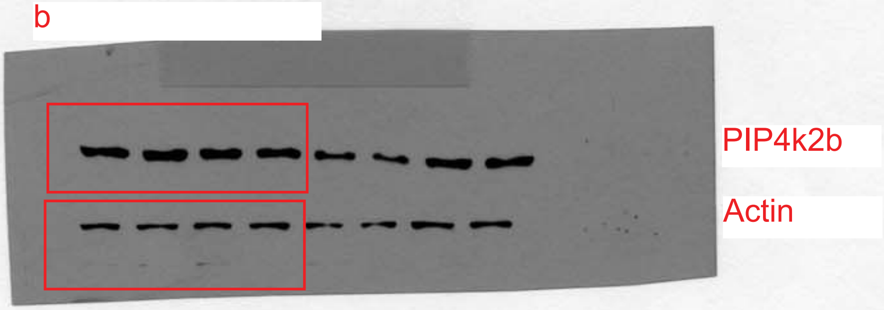

Supplementary Figure 11 (continued)

Supplementary Figure 9

a

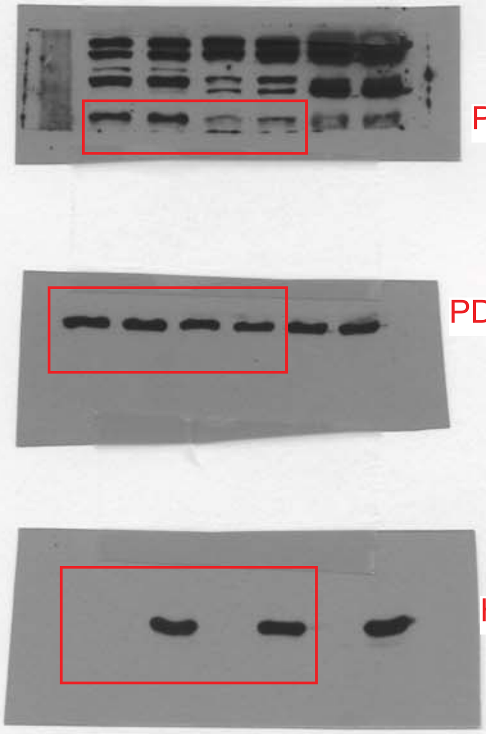

PIP4k2b

PDI

HA

c

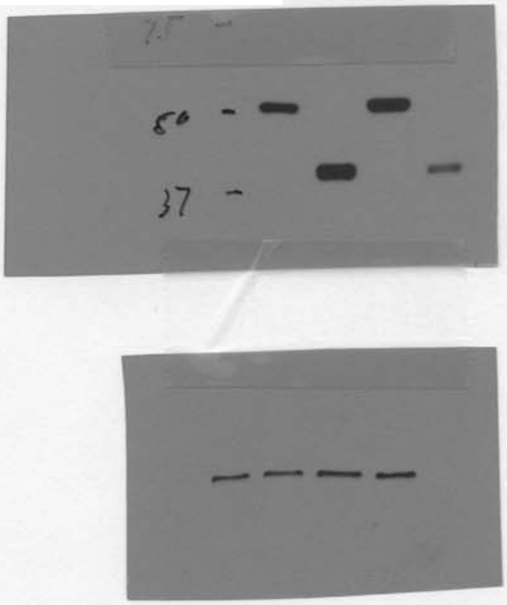

Myc

PDI

d

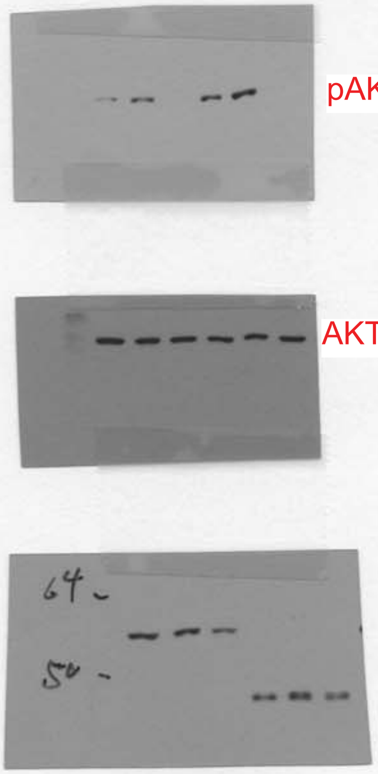

pAKT

AKT

Myc

Supplementary Figure 11 (continued)

Supplementary Figure 10

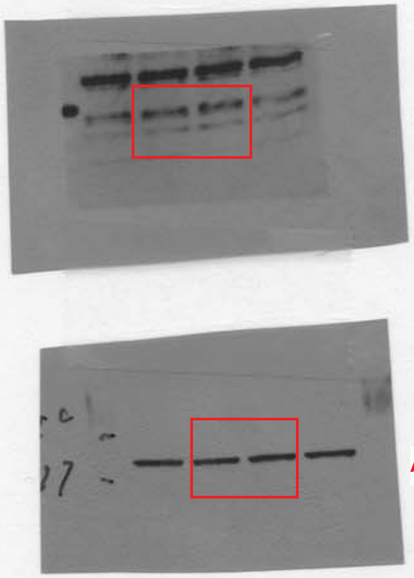

Supplementary Figure 11 Uncropped western blot images
